# *Cladophialophora carrionii* [kladˊ-ō-fī-əl-ō-for-ə karˊ-ē-ō-nē-ī]

**DOI:** 10.3201/eid3111.240204

**Published:** 2025-11

**Authors:** Dayane Moraes, Alexandre Melo Bailão, Mirelle Garcia Silva Bailão

**Affiliations:** Universidade Federal de Goiás (UFG), Goiânia, Brazil

**Keywords:** Cladophialophora carrionii, fungi, chromoblastomycosis, Arturo I. Carrión

*Cladophialophora carrionii* is a black fungus that causes chromoblastomycosis. The fungus was first reported in 1946 as *Fonsecaea pedrosoi* var. *cladosporium*. In 1954, mycologist Alfonso Trejos named this chromoblastomycosis agent *Cladosporium carrionii* n. sp. (Figure). Later, molecular phylogenetic analysis and morphologic traits (cladosporioid anamorphs and phialidic synanamorphs) supported its current placement in the genus *Cladophialophora*. 

**Figure:**
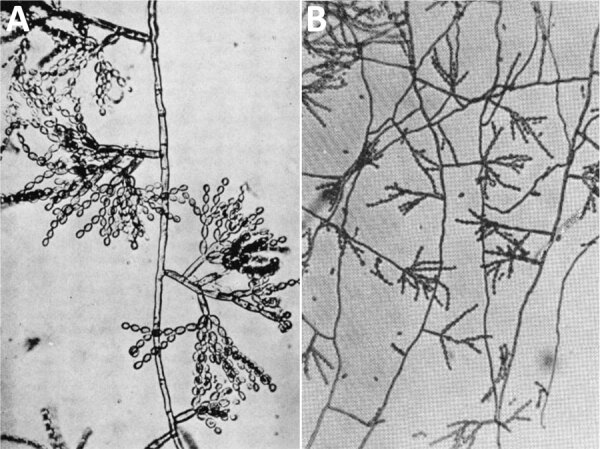
Images from early records of *Cladosporium carrionii*. A) *Fonsecaea pedrosoi* var. c*ladosporium* by F.W. Simson in 1946. B) *Cladosporium carrionii* n. sp. by A. Trejos in 1954.

The term *Cladophialophora* is derived from the Greek words *klados* (branch) and *phiala* (flask or bottle) and the suffix *phora*, which means bearing or carrying, emphasizing that these structures produce and harbor the fungal spores. The specific epithet *carrionii* is in honor of the charismatic Puerto Rican mycologist Prof. Dr. Arturo I. Carrión, renowned for his contributions to medical mycology, particularly in the taxonomy and clinical understanding of dematiaceous fungal pathogens. Therefore, the full name *Cladophialophora carrionii* means a black flask-shaped fungus with branched conidia chains, named in honor of Dr. Carrión.

## References

[1] Bensch K, Braun U, Groenewald JZ, Crous PW. The genus *Cladosporium.* Stud Mycol. 2012;72:1–401. 10.3114/sim0003 22815589 PMC3390897

[2] de Hoog GS, Guého E, Masclaux F, Gerrits van den Ende AH, Kwon-Chung KJ, McGinnis MR. Nutritional physiology and taxonomy of human-pathogenic *Cladosporium-Xylohypha* species. J Med Vet Mycol. 1995;33:339–47. 10.1080/02681219580000661 8544087

[3] Simson FW. Chromoblastomycosis; some observations on the types of the disease in South Africa. Mycologia. 1946;38:432–49. 10.1080/00275514.1946.12024066 20992395

[4] Trejos A. *Cladosporium carrionii* n. sp. and the problem of *Cladosporia* isolated from chromoblastomycosis. Rev Biol Trop. 1954;2:75–112.

